# Integrated physiological and transcriptomic analysis reveals the key pathways of *Rosa rugosa* in response to salt-alkali stress

**DOI:** 10.3389/fpls.2025.1679259

**Published:** 2025-12-01

**Authors:** Lulu Han, Yan Lu, Mengxin Niu, Meiying Liu, Kebin Yang

**Affiliations:** 1 Key Laboratory of Biochemistry and Molecular Biology in University of Shandong, School of Advanced Agricultural Sciences, Weifang University, Weifang, China; 2 College of Landscape Architecture and Forestry, Qingdao Agricultural University, Qingdao, China

**Keywords:** *Rosa rugosa*, salt-alkali stress, physiological mechanism, transcriptome, phenylpropanoid biosynthesis

## Abstract

**Introduction:**

Abiotic stressors, particularly saline–alkali stress, restrict plant growth and development. *Rosa rugosa*, which grows in coastal areas and exhibits high saline–alkali tolerance, serves as an ideal model for analyzing rose response mechanisms to saline–alkali stress (SAS). However, its response mechanisms have not yet been elucidated.

**Methods:**

This study examined SAS using a 150 mmol·L^−1^ saline–alkali solution and analyzed the physiological and molecular response mechanisms using physiological and biochemical indicators and high-throughput RNA-sequencing technology.

**Results:**

Under SAS, reactive oxygen species accumulation increased, resulting in extensive oxidative damage to cell membranes. In response, the superoxide dismutase, peroxidase, and catalase activities, along with the contents of soluble sugars, soluble proteins, and proline increased. Furthermore, 325, 2,197, 4,266, and 6,842 differentially expressed genes (DEGs) were identified at 6, 12, 24, and 48 h of SAS, respectively. Functional annotation and pathway enrichment analyses indicated that DEGs were primarily involved in cell wall organization, enzyme activity, biosynthesis of secondary metabolites, and photosynthesis pathways. Several structural genes from the phenylpropanoid biosynthesis pathway, including *PAL*, *4CL*, *HCT*, *CCR*, *COMT*, *CHS*, *CHI*, and *DFR*, were identified by qRT-PCR, which positively responded to SAS and peaked at 12 h. Weighted gene co-expression network analysis revealed that *PKS* likely functions as the hub gene in the secondary metabolic pathway responding to SAS.

**Discussion:**

This study advances understanding of saline–alkali resistance mechanisms, and the identified genes and metabolic pathways can enhance future rose breeding efforts.

## 1 Introduction

Soil salinization–alkalization has become a major limiting factor in agriculture and forestry development (Shabala et al., 2014; Munns et al., 2020). According to a report by the Food and Agriculture Organization of the United Nations, approximately 9.6 × 10^8^ ha of land worldwide experiences varying degrees of salinization and alkalinization, and this area continues to expand (Munns et al., 2020). In China, nearly 1.0 × 10^8^ ha of saline–alkali land exists, of which 10% has development and utilization potential. Therefore, understanding the saline–alkali stress (SAS) response mechanisms in plants, identifying saline–alkali resistance functional genes, and using plant cultivation to achieve ecological improvement of saline–alkali land are important.

Rose (*Rosa rugosa*) is a deciduous shrub native to Eastern Asia with high ornamental value. Its flowers contain diverse compounds, including primary and secondary metabolites (Chen et al., 2021), and are widely used in the food industry as spice and in the cosmetics industry for their essential oils (Li et al., 2021; Cui et al., 2022). However, saline–alkali tolerance is neglected during flower trait breeding of most rose cultivars, resulting in limited plantation despite abundant saline–alkali soil areas in China (Zang et al., 2021). Screening identified that coastal wild rose is capable of thriving in saline–alkali soil with 0.6–1% soil salt concentration and approximately 9.5 pH value (**Supplementary Figure S1**), representing an ideal saline–alkali tolerant germplasm resource for understanding the saline–alkali tolerance mechanism.

In addition to inducing osmotic stress and ion toxicity by disrupting ion homeostasis and balance in plant cells, SAS, as two coexisting abiotic stresses, triggers oxidative stress and high pH stress (Fang et al., 2021; Wang et al., 2021), which destroys plant cell structure, leading to membrane lipid peroxidation and protease activation (Tsikas, 2017). Based on this, plants have developed multiple mechanisms and pathways to coordinately respond to SAS. In plants, the synthesis and accumulation of numerous small organic molecules alter the solvent properties of water to stabilize intracellular osmotic pressure and protect macromolecular structures. Similar findings have been documented in *Sorghum bicolor* (Sun et al., 2019), *Oryza sativa* (Xu et al., 2024), *Medicago sativa* (Bhattarai et al., 2020), *Betula platyphylla* (Mijiti et al., 2017), *Nitraria billardieri* (Tian et al., 2018), and *Chenopodium quinoa* (Li and Zhang, 2025), which accumulate substantial amounts of osmotic adjustment substances, such as proline, betaine, soluble sugars, and organic acids, under SAS, maintaining charge homeostasis and regulating osmotic adjustment substances within the plant to stabilize cells. Furthermore, certain osmotic adjustment substances function as signals, inducing increases in endogenous hormone content that affect the expression of related genes, thereby regulating plant growth under salt stress (Marusig and Tombesi, 2020; Zhao et al., 2021).

Plants typically activate the reactive oxygen species (ROS) scavenging system, which includes both enzymatic and non-enzymatic scavenging mechanisms, to counteract the oxidative damage caused by excessive ROS accumulation. Among these, the enzymatic scavenging system comprises superoxide dismutase (SOD), peroxidase (POD), and catalase (CAT), which collectively eliminate malondialdehyde (MDA) produced by lipid peroxidation to protect membrane structures (Kapoor et al., 2019; Sanoubar et al., 2020). In rice, *OsLOL5* overexpression induces genes related to oxidative stress, such as *OsAPX2*, *OsCAT*, *OsCu/Zn-SOD*, and *OsRGRC2*, thereby enhancing saline–alkali tolerance through the ROS detoxification pathway (Guan et al., 2016). The non-enzymatic antioxidant system consists of flavonoids, anthocyanins, alkaloids, and carotenoids, whose production can also reduce ROS accumulation (Zhang et al., 2011). Ben Abdallah et al. (2016) demonstrated that secondary metabolites improve plant salt tolerance by scavenging ROS and regulating cell osmotic pressure.

In response to SAS, maintaining Na^+^ balance is particularly crucial for plants. Plants primarily reduce cytoplasmic Na^+^ accumulation by promoting Na^+^ efflux and compartmentalization (Zhao et al., 2020). Na^+^ efflux occurs by the salt overly sensitive signaling pathway, and its role in saline–alkali resistance, which is sensing Ca^2+^ signals to expel excess Na^+^ from cells (Quan et al., 2007; Lin et al., 2009), has been confirmed in maize (Zhou et al., 2022) and tomato (Yang et al., 2022). Na^+^ compartmentalization into vacuoles is another strategy used by plants to reduce Na^+^ toxicity. In this process, NHX, H^+^-ATPase, and high-affinity K+ transporter serve as crucial regulators that enhance plant tolerance by modulating the dynamic balance of Na^+^, H^+^, and K^+^ under SAS (Batelli et al., 2007; Chen et al., 2015; Zhang et al., 2021).

RNA-sequencing (RNA-seq) analysis has revealed numerous abiotic stress response pathways and functional genes across plant species. In *Nicotiana tabacum*, ethylene response factor (ERF) *ERF13a* directly binds to the GCC box or dehydration-responsive element *cis*-acting element of hydroxycinnamoyl transferase (*HCT*), flavanone 3-hydroxylase (*F3H*), and anthocyanidin synthase (*ANS*), promoting phenylpropanoid accumulation and thereby enhancing plant tolerance to abiotic stress (Wang et al., 2023). In *Leymus chinensis*, the phenylalanine metabolic pathway and the citrate cycle pathway work synergistically to induce organic acid secretion and mitigate heavy metal pollution (Guan et al., 2024). In *Lolium perenne*, the phenylpropanoid biosynthesis pathway serves as the main secondary metabolic pathway responding to salt stress, with salt-tolerant lines exhibiting higher levels of phenylpropanoids, flavonoids, anthocyanins, and related gene expression compared with sensitive lines (Cao et al., 2024). Furthermore, RNA-seq analysis has identified multiple candidate saline–alkali tolerant transcription factors (TFs) suitable for genetic editing, including ERF (Jiang et al., 2024), WRKY (Qin et al., 2015), basic helix–loop–helix (bHLH) (Ahmad et al., 2015), and basic leucine zipper (bZIP) (Xu et al., 2023), which play essential roles in regulating structural genes and metabolic pathways, thus helping elucidate the molecular mechanisms of plant responses to SAS.

The molecular mechanisms of SAS tolerance have been extensively studied in model plants and agricultural crops, including *O. sativa* (Wang et al., 2024), *Avena sativa* (Gao et al., 2022), *M. sativa* (Hang et al., 2024), and *Malus halliana* (Jia et al., 2019). However, a comprehensive investigation of abiotic stress adaptation mechanisms in ornamental plants remains relatively limited, particularly under conditions of high salinity and alkalinity.

This study examines rose species with high saline–alkali resistance to elucidate their physiological response mechanisms to SAS through the analysis of physiological and biochemical indicators. Using RNA-seq technology, the key response pathways and regulatory factors of SAS were investigated through molecular biology methods including differentially expressed gene (DEG) screening, enriched pathway analysis, and quantitative real-time polymerase chain reaction (qRT-PCR) validation. The research aims to enhance understanding of the molecular mechanisms underlying rose responses to SAS, expand the repertoire of saline–alkali resistance functional genes, and establish a theoretical foundation for breeding high-quality saline–alkali tolerant varieties.

## 2 Materials and methods

### 2.1 Plant materials and treatments

The experimental materials used in this study were wild roses cultivated in coastal areas, and the experiment was conducted at Weifang University (Shandong, China). One-year-old cutting seedlings of rose plants exhibiting uniform growth were selected and maintained under common greenhouse conditions for 30 days. Healthy seedlings were subsequently transferred to boxes containing clean water (pH = 6.8 ± 0.2) with roots submerged. Three seedlings were cultivated per box across 24 boxes in total, with continuous oxygen ventilation throughout the cultivation period. Following a 7-day adaptation period, 12 boxes were selected for SAS treatment (T), involving exposure to 150 mmol·L^−1^ saline–alkali solution (NaCl: NaHCO_3_ = 1:1 volume ratio) (pH = 8.5). The remaining 12 boxes continued receiving clean water (CK). Leaves from corresponding positions under both treatment and control groups were collected at 6 h (T1 and CK1), 12 h (T2 and CK2), 24 h (T3 and CK3), and 48 h (T4 and CK4), and stored at −80°C after rapid freezing in liquid nitrogen for physiological parameter measurement, transcriptome sequencing, and qRT-PCR validation. Each treatment was repeated three times, with three biological replicates per sample per experimental repetition.

### 2.2 Histochemical staining and physiological parameter measurement

The collected rose leaves were washed and cleaned, and histochemical staining was performed using diaminobenzidine (DAB) and trypan blue staining kits (Solarbio, Beijing, China).

Rose leaves were harvested for physiological parameter measurement. The SOD, POD, and CAT activities, along with the contents of H_2_O_2_, proline, MDA, soluble sugars, and soluble proteins, were measured using appropriate detection kits (Solarbio, Beijing, China).

### 2.3 RNA extraction, library construction, sequencing and transcriptomic analysis

RNA-seq analysis was performed by Novogene Bioinformation Technology Co., Ltd. (Beijing, China). Total RNA was extracted from rose leaves using an RNA extraction kit (TRIzol; Tiangen, Beijing, China). RNA purity and concentration were assessed using 1% agarose gel electrophoresis and NanoDrop2100 (Agilent, USA), with RIN scores and electrophoretograms ultimately provided to confirm the suitability of samples for sequencing. The cDNA library was constructed using a Fast RNA-seq Lib Prep Kit V2 (ABclonal, Wuhan, China). mRNA was enriched using oligo (dT) beads and fragmented with Frag/Elute buffer. First and second strand cDNAs were synthesized with random hexamer primers. The cDNAs were then given a poly(A) tail and P5 adaptors were added. cDNA fragments ranging from 370 to 420 bp were selected and followed by PCR amplification. The quality of the libraries was assessed using an Agilent 2100 bioanalyzer. A total of 24 prepared libraries underwent high-throughput sequencing using an Illumina NovaSeq 6000. High-quality clean reads were selected and low-quality reads were filtered using fastp software. The reference genome index (https://ngdc.cncb.ac.cn/gwh/Assembly/30680/show) was constructed using HISAT2 (v2.0.5), and paired-end clean reads were aligned to the reference genome using HISAT2. Gene expression was quantified using the Fragments per Kilobase per Million mapped reads (FPKM) method.

### 2.4 Correlation analysis

Based on transcriptome data, antioxidant enzyme-related DEGs were identified using the criterion: FPKM value ≥ 2 across all four periods under SAS. The correlation between antioxidant enzyme-related gene expression levels and antioxidant enzyme activities was evaluated using the Pearson coefficient method. Higher absolute values indicate stronger correlations, with positive and negative values representing positive and negative correlations, respectively.

### 2.5 DEGs screening and functional enrichment analysis

DEGs were identified between samples using the DESeq2 R package (v1.20.0), which provides statistical tools for detecting differential expression in digital gene expression datasets using models based on the negative binomial distribution. The resulting *p*-value was adjusted using the Benjamini–Hochberg method to control the false discovery rate. Significant differential expression was defined by a corrected *p*-value ≤ 0.05 and |log_2_(foldchange)| ≥ 2. Gene Ontology (GO) and Kyoto Encyclopedia of Genes and Genomes (KEGG) enrichment analyses of DEGs were performed using the clusterProfiler R package.

### 2.6 Total RNA extraction and reverse transcription

Total RNA was extracted from rose leaves using the FastPure Universal Plant Total RNA Isolation Kit (Vazyme, Nanjing, China). RNA integrity and concentration were assessed using 1.0% agarose gel electrophoresis and an Agilent 2100 Bioanalyzer (Agilent Technologies, Santa Clara, CA, USA). The cDNA library was synthesized using TransScript^®^ First-Strand cDNA Synthesis SuperMix (TransGen Biotech, Beijing, China). Samples were stored at −20°C for sequencing.

### 2.7 The qRT-PCR validation

The qRT-PCR analysis was performed using ChamQ Universal SYBR qPCR Master Mix (Vazyme) on a LightCycler 480 system (Roche, Mannheim, Germany). Experimental procedures followed the manufacturer’s instructions. *GAPDH* served as a reference gene (Zang et al., 2024). The 2^−△△CT^ method was used for analysis and visualization of qRT-PCR data from multiple technical replicates. Specific primer information is provided in **Supplementary Table S8**.

### 2.8 Weighted gene co-expression network analysis (WGCNA)

WGCNA was conducted using the R package WGCNA v1.71. FPKM values were analyzed using the blockwiseModules function with default parameters, except for soft-thresholding power of 10, mergeCutHeight of 0.8, minModuleSize of 50, and unsigned TOMType. Co-expression networks and genes within the MEred module, with edge weights exceeding 0.15, were visualized using Cytoscape (v3.9.1).

### 2.9 Statistical analysis

All experimental data were visualized and analyzed using one-way analysis of variance in GraphPad Prism (v10.5.0). Differences of *P* < 0.05 were considered significant. The experimental results are presented as the mean ± standard deviation of three replicates.

## 3 Results

### 3.1 Determination of physiological and biochemical indicators of rose under SAS

**Figure 1:**
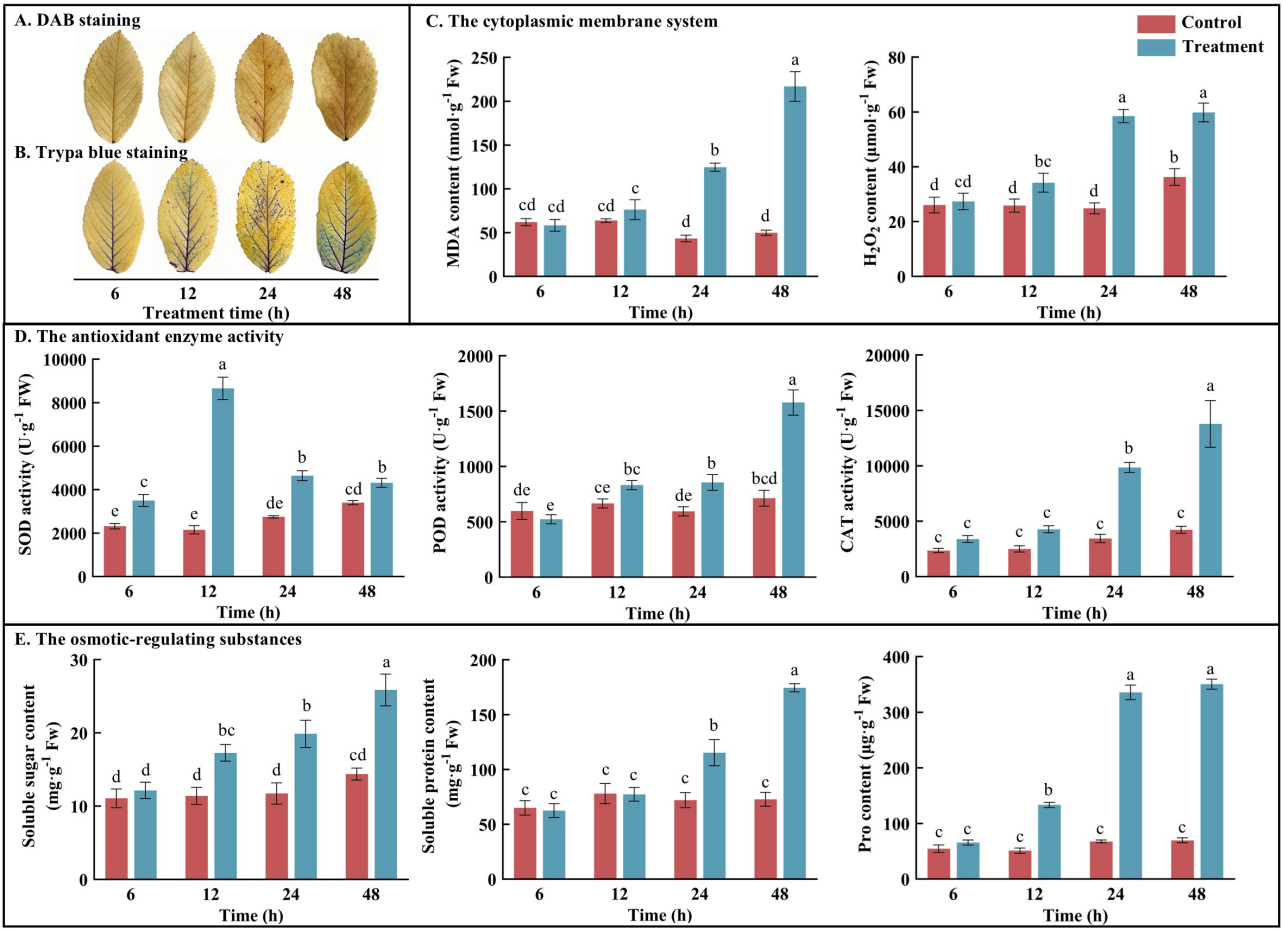
Effect of different durations of SAS on **(A)** DAB staining; **(B)** Trypa blue staining; **(C)** The content of MDA and H_2_O_2_; **(D)** The activity of SOD, POD and CAT; **(E)** The content of soluble sugar, soluble protein and proline. Data are the means (± SD) from three biological replicates. For the same parameter, different letters above the bars indicate a significant difference at P<0.05 level. The control and treatment represent clear water and 150 mmol·L^-1^ saline-alkali solution, respectively. Each treatment was performed using at least three biological replicates.

To investigate the effects of SAS on rose, ROS production was monitored by H_2_O_2_ and MDA measurements, while cell H_2_O_2_ accumulation and cell membrane damage were visualized by DAB and trypan blue histochemical staining procedures, respectively. As SAS duration increased, H_2_O_2_ and MDA contents in rose leaves showed an overall increasing trend (**Figure 1C**), with DAB and trypan blue staining intensifying progressively (**Figures 1A, B**), displaying significant coloration after 24 h. During all four periods of clean water treatment (CK1–4), no significant variations were observed in physiological indicators and staining results (**Supplementary Figure S2**). These observations suggest that SAS induced excessive ROS production, resulting in substantial membrane lipid peroxidation. Thus, the antioxidant enzyme activities (SOD, POD, and CAT) and osmotic adjustment substances (proline, soluble sugars, and soluble proteins) in rose leaves increased markedly. Compared with the control, most indicators exhibited significant differences (*P* < 0.05) after 12 h of saline–alkali treatment, suggesting that SAS activated ROS scavenging and osmotic adjustment systems, which are crucial for maintaining membrane integrity and cellular functions.

### 3.2 Transcriptomic analysis of rose leaves

**Figure 2:**
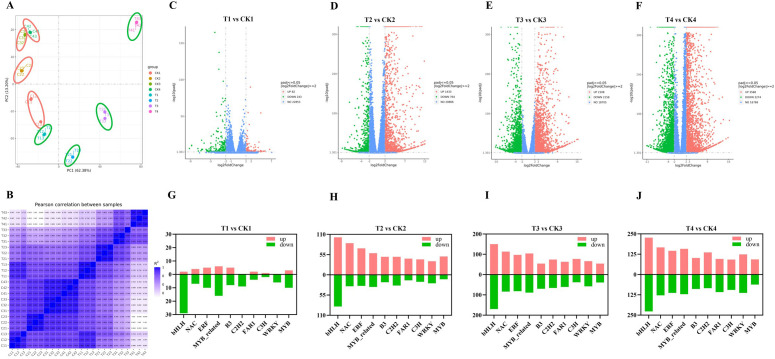
Analysis of DEGs in the process of SAS. **(A)** PCA; **(B)** Pearson correlation between samples; **(C–F)** Volcano maps for different comparison groups; **(G–J)** Statistics of DEGs in TF families. The red and green bars indicate the number of upregulated and downregulated genes, respectively.

To elucidate the detailed molecular mechanism of rose response to SAS, transcriptome analysis was performed on control and treatment group samples. The analysis generated 171.92 G of raw data, yielding 167.41 G of clean data, with Q20 and Q30 values exceeding 98.07% and 95.09%, respectively, and GC content above 42.95% (**Supplementary Table S1**). Principal component analysis results revealed distinct grouping and separation between treatments, demonstrating high data accuracy and authenticity, with strong within-group repetition (**Figures 2A, B**). These results confirmed the high quality of assembled transcripts.

The analysis yielded 35,903 clean reads, and comparative analysis of T1 vs. CK1, T2 vs. CK2, T3 vs. CK3, and T4 vs. CK4 identified 325 (82 upregulated and 243 downregulated), 2,197 (1,433 upregulated and 764 downregulated), 4,266 (2,108 upregulated and 2,158 downregulated), and 6,842 (3,568 upregulated and 3,274 downregulated) DEGs, respectively (|log2(foldchange)|≥2 and corrected *p*-value ≤ 0.05) (**Figures 2C–F**; **Supplementary Table S2**). Notably, DEG numbers increased substantially after 12 h of SAS, corresponding to physiological indicator changes. These findings indicate significant SAS impact on gene expression, demonstrating dynamic transcriptional regulation in response to SAS in rose.

Statistical analysis of DEG numbers between treatment and control groups revealed that TFs constituted a large proportion, particularly after 12 h. T2 vs. CK2 comparison identified 188 bHLHs, 117 NACs, 102 ERFs, 92 MYB_related, and 69 B3s and others, comprising 102, 86, 72, 59, and 49 upregulated and 86, 31, 30, 33, and 20 downregulated genes, respectively. T3 vs. CK3 comparison revealed 320 bHLHs, 197 NACs, 193 MYB_related, 179 ERFs, and 140 C2H2s and others, including 150, 113, 104, 97, and 74 upregulated and 170, 84, 89, 82, and 66 downregulated genes. T4 vs. CK4 comparison showed 454 bHLHs, 295 NACs, 279 MYB_related, 259 ERFs, and 237 WRKYs and others, with 227, 167, 158, 146, and 124 upregulated and 227, 128, 121, 113, and 113 downregulated genes, respectively (**Figures 2G–J**; **Supplementary Table S3**). These results highlight the dynamic transcriptional regulation under varying conditions.

### 3.3 Correlation analysis between gene expression pattern and enzyme activity

**Figure 3:**
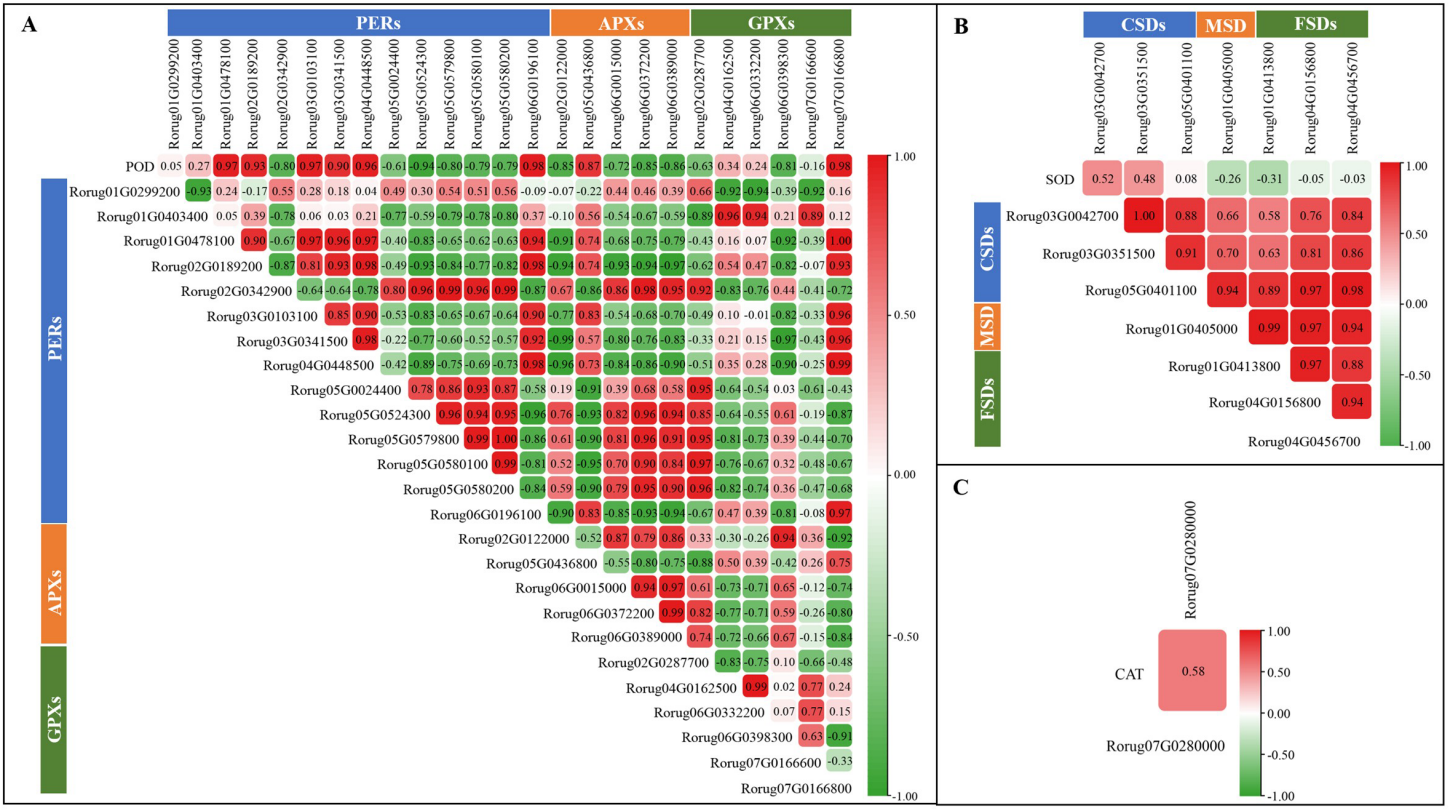
Analysis of the correlation between antioxidant enzyme-related genes expression patterns and the antioxidant enzymes activities. **(A)** POD; **(B)** SOD; **(C)** CAT. The numbers in the rectangle represent the correlation coefficient. The higher the absolute value implies the more correlated, and the positive and negative values represent positive and negative correlations, respectively.

The antioxidant enzyme system, functioning as the primary plant defense mechanism against abiotic stress, maintains cellular redox homeostasis by effectively eliminating ROS, thus ensuring plant survival and development. This study identified seven *SOD* genes, including three *Cu/Zn-SOD*s (*CSD*s), one *Mn-SOD* (*MSD*), and three *Fe-SOD*s (*FSD*s); 25 *POD* genes, comprising 14 class III secretory PODs (*PER*s/*PRX*s), five ascorbate PODs (*APX*s), and six glutathione PODs (*GPX*s); and one CAT-related gene. Notably, the expression patterns of six *PER*s (Rorug01G0478100, Rorug02G0189200, Rorug03G0103100, Rorug03G0341500, Rorug04G0448500, and Rorug06G0196100) demonstrated correlation coefficients exceeding 0.9 with POD activity, while one *APX* (Rorug05G0436800) and one *GPX* (Rorug07G0166800) exhibited correlation coefficients of 0.87 and 0.98 with POD activity, respectively (**Figure 3A**). Furthermore, correlation analysis between SOD-related gene expression patterns and SOD activity revealed that *CSD* subclass genes showed positive correlation, with Rorug03G0042700 demonstrating the highest correlation coefficient. Conversely, both *MSD* and FSD members showed negative correlations (**Figure 3B**). A single CAT-related gene was identified, with its expression pattern showing a correlation coefficient of 0.58 with CAT activity (**Figure 3C**). These findings indicate that antioxidant enzyme activity increases by the regulation of antioxidant enzyme-related gene expression, thereby enhancing ROS scavenging capacity in rose.

### 3.4 Analysis of overlapped DEGs among different groups

**Figure 4:**
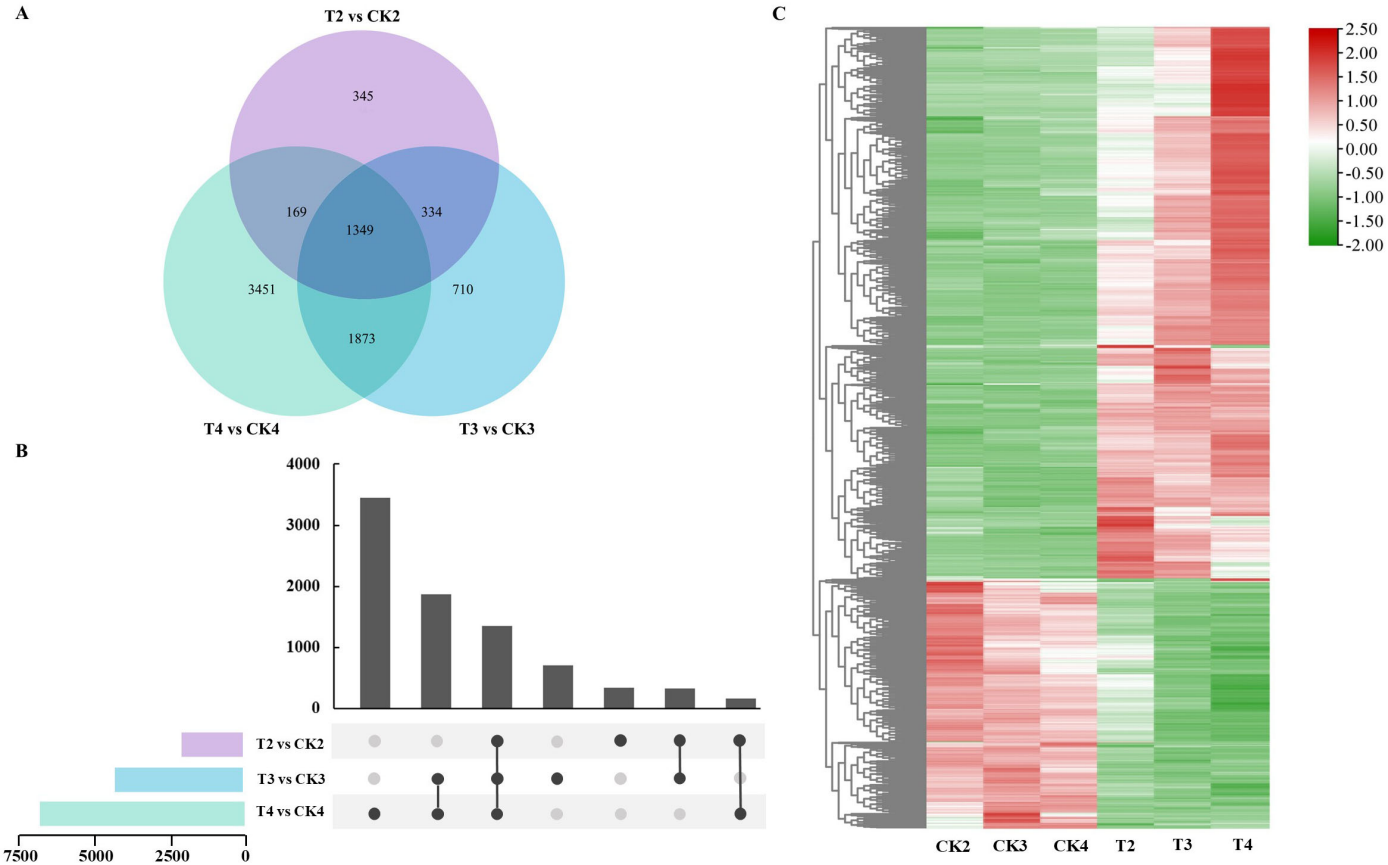
Profiling of overlapped DEGs among different control and treatment groups. **(A)** Venn diagrams of DEGs; **(B)** Upset bar graphs of DEGs; **(C)** Heatmap visualization and clustering of overlapped DEGs among three groups. The color from green to red indicating low to high expression abundance.

Given the limited number of DEGs at 6 h (T1 vs. CK1), which could affect subsequent analysis of overlapping DEGs, this section focuses on three time points: 12 h (T2 vs. CK2), 24 h (T3 vs. CK3), and 48 h (T4 vs. CK4). Statistical analysis of overlapping DEGs between treatment groups revealed 345, 710, and 3,451 unique DEGs in T2 vs. CK2, T3 vs. CK3, and T4 vs. CK4, respectively. Overlapping DEGs between pairs of groups were 334 (T2 vs. CK2 and T3 vs. CK3), 169 (T2 vs. CK2 and T4 vs. CK4), and 1,873 (T3 vs. CK3 and T4 vs. CK4) (**Figures 4A, B**; **Supplementary Table S4**). A total of 1,349 DEGs overlapped among the three groups. Further analysis of co-expressed gene expression patterns during SAS demonstrated that the majority of overlapping DEGs was upregulated under SAS, exhibiting diverse expression trends with extended stress duration. Thus, most genes in rose respond to SAS through complex transcriptional regulation mechanisms.

### 3.5 Functional annotation analysis of DEGs

**Figure 5:**
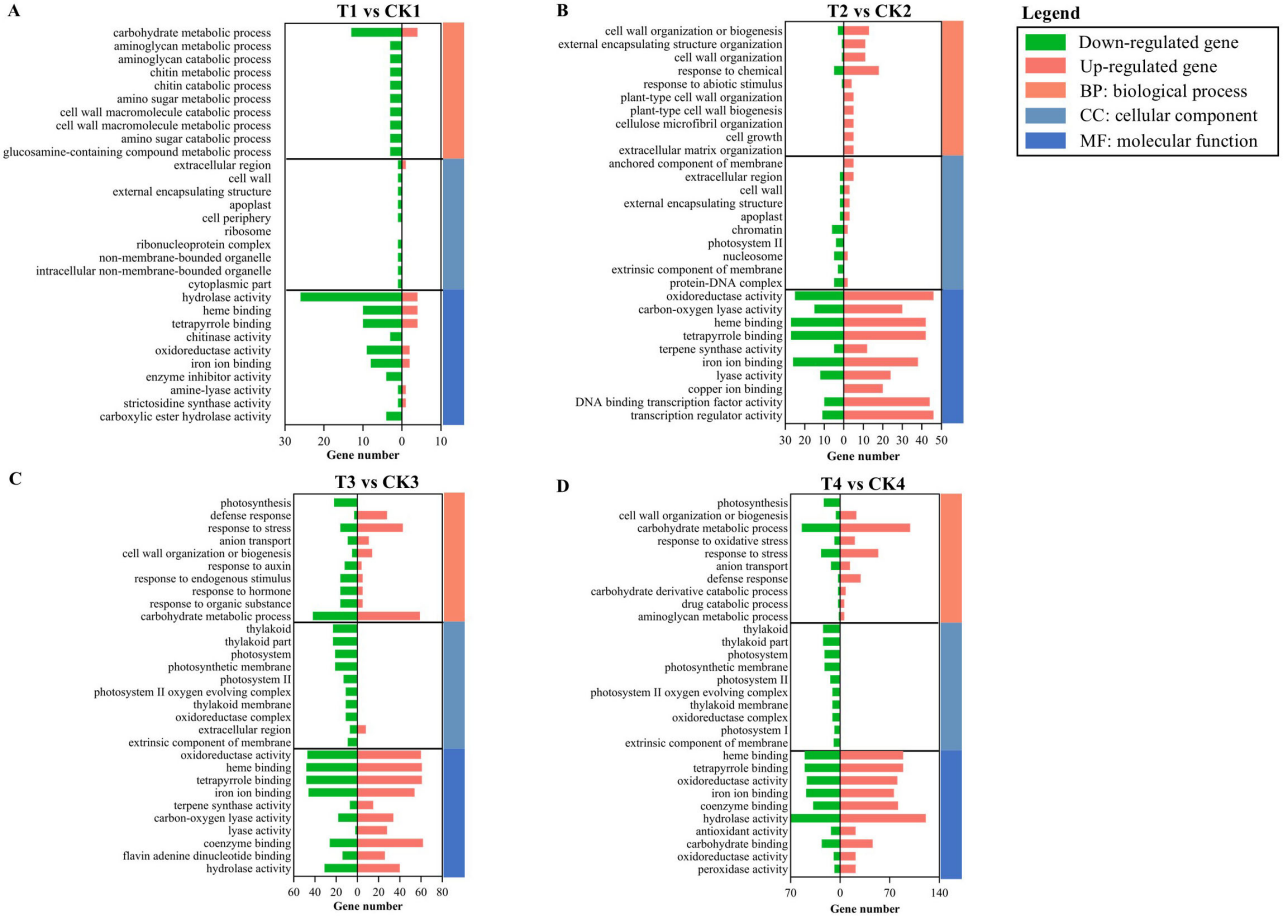
GO functional classification of the DEGs during SAS. **(A)** 6 h-SAS/CW; **(B)** 12 h-SAS/CW; **(C)** 24 h-SAS/CW; **(D)** 48 h-SAS/CW. Green represent the down-regulated genes, red represent the up-regulated gene.

The GO analysis results included all three categories: biological process, molecular function, and cellular component. Statistical analysis focused on the top 10 pathways from each GO category (**Supplementary Table S5**). In T1 vs. CK1, DEGs were limited, with almost all showing downregulation (**Figure 5A**). From T2 vs. CK2 onward, the number of DEGs enriched in pathways significantly increased. Specifically, upregulated DEGs were mainly enriched in “cell wall organization or biogenesis” and “response to chemical” of the biological process category, while “oxidoreductase activity”, “DNA-binding transcription factor activity”, and “transcription regulator activity” of the molecular function category were prominent (**Figure 5B**). Comprehensive analysis of T3 vs. CK3 and T4 vs. CK4 revealed similar patterns: at the biological process level, both upregulated and downregulated DEGs were enriched in pathways related to metabolic metabolism and responses; regarding cellular component, except for eight upregulated genes enriched in “extracellular region” in T3, all other pathways contained exclusively downregulated DEGs; and in molecular function, DEGs were enriched in the functional categories associated with enzyme activity and binding (**Figures 5C, D**).

**Figure 6:**
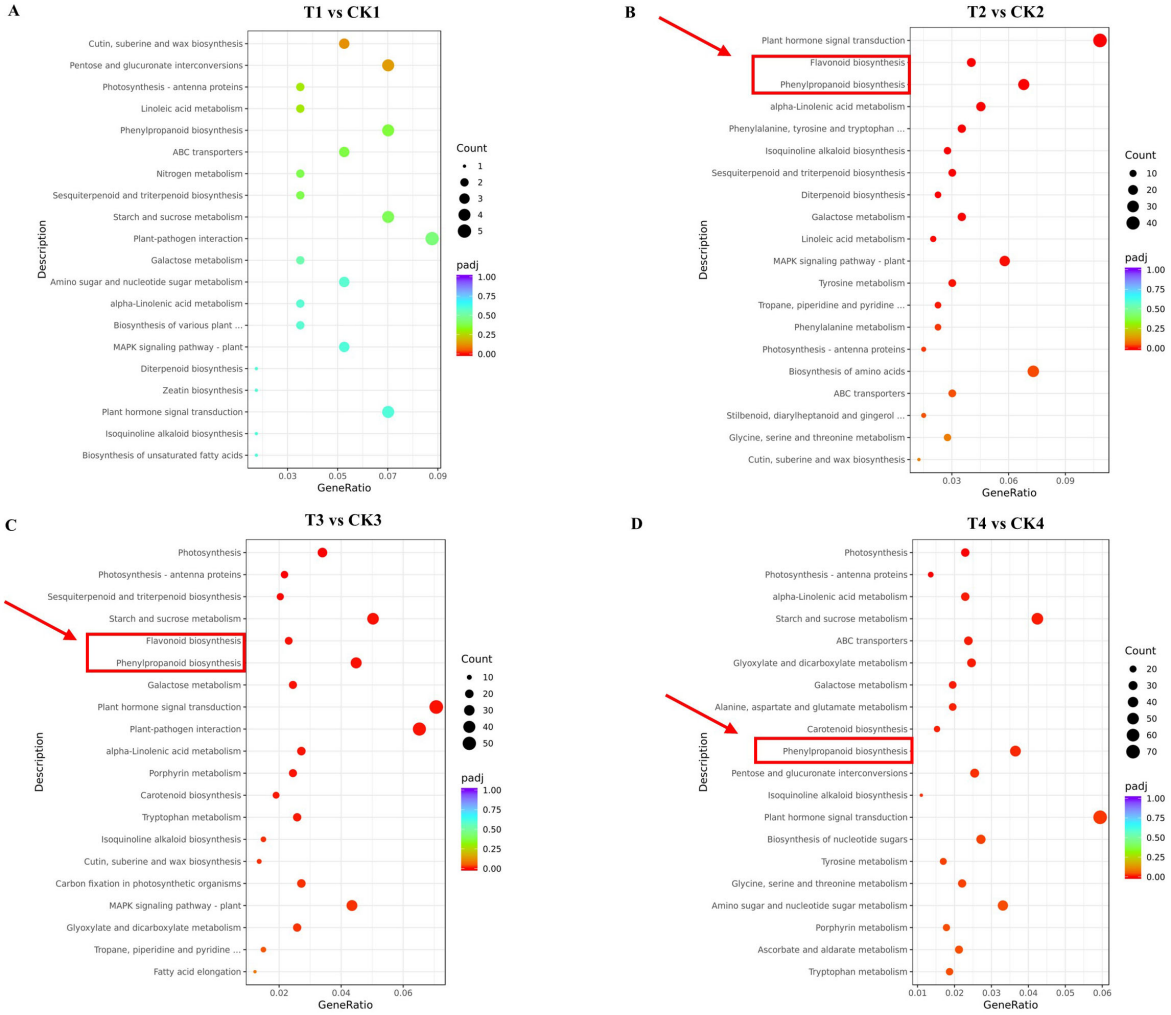
KEGG enrichment analysis of DEGs in top20 during SAS. **(A)** 6 h-SAS/CW; **(B)** 12 h-SAS/CW; **(C)** 24 h-SAS/CW; **(D)** 48 h-SAS/CW. The sizes of the spots represent the number of genes and the colors represent the *p*-value in each pathway.

The top 20 results of KEGG enrichment analysis are presented in **Supplementary Table S6**. In T1 vs. CK1, KEGG pathways showed no significant over-representation in either enriched gene set (**Figure 6A**), likely because of the limited number of genes. However, in T2 vs. CK2, the altered pathways focused on “plant hormone signal transduction”, “flavonoid biosynthesis”, and “phenylpropanoid biosynthesis” (**Figure 6B**). The pathways of “photosynthesis”, “photosynthesis - antenna proteins”, and “starch and sucrose metabolism” were significantly enriched in T3 vs. CK3 and T4 vs. CK4. In addition, “flavonoid biosynthesis” and “phenylpropanoid biosynthesis” showed notable enrichment in T3 vs. CK3 (**Figure 6C**), while “ABC transporters”, “carotenoid biosynthesis”, and “phenylpropanoid biosynthesis” were enriched in T4 vs. CK4 (**Figure 6D**). Notably, “phenylpropanoid biosynthesis” emerged as a common pathway at three key nodes (T2, T3, and T4), and “flavonoid biosynthesis” appeared as a common pathway at two key nodes (T2 and T3). This suggested that these pathways are crucial for rose response to SAS.

### 3.6 Analysis of DEGs related to phenylpropanoid biosynthesis pathways

**Figure 7:**
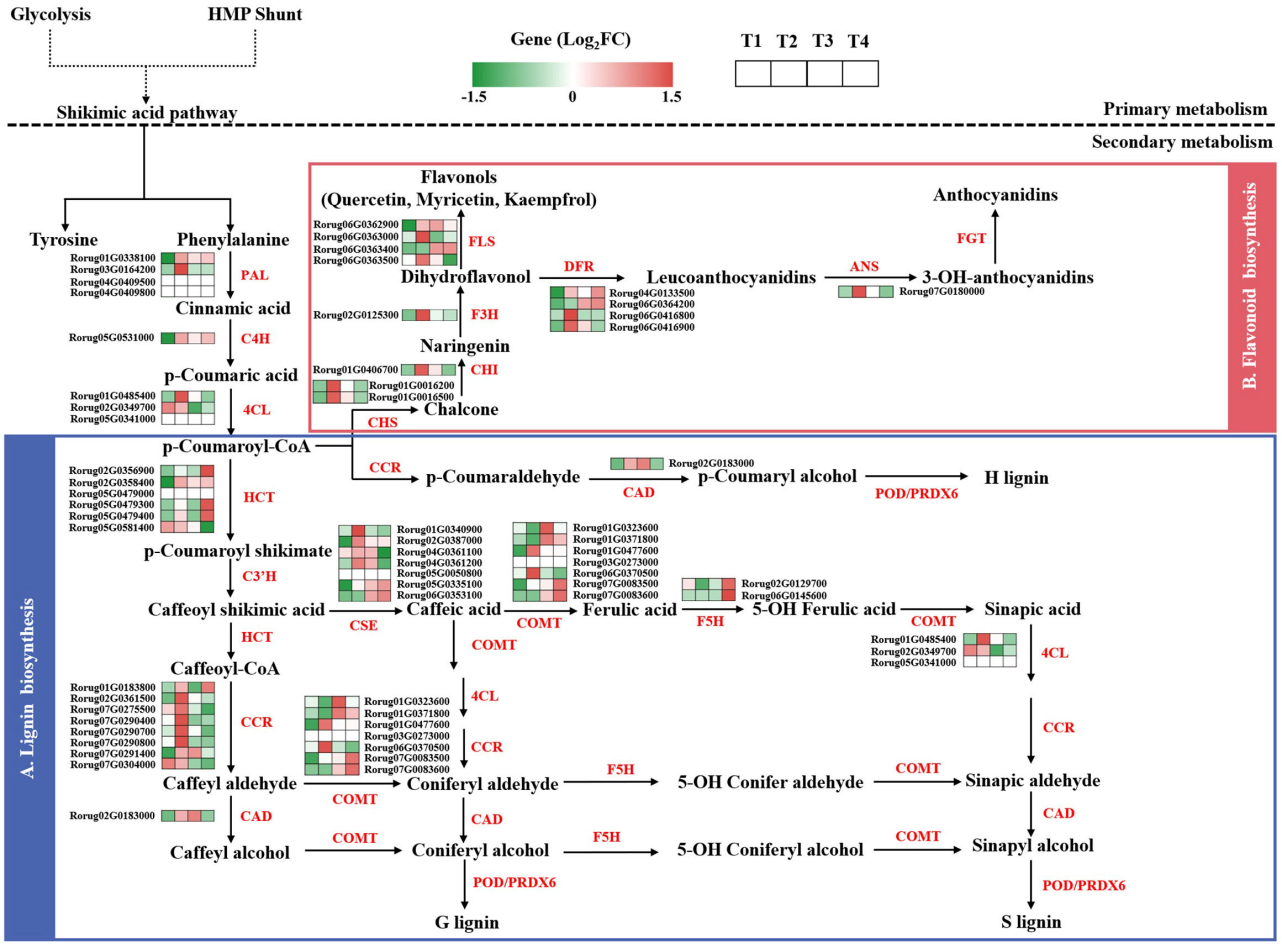
The phenylpropanoid biosynthesis pathways of rose. Expression abundance of DEGs in **(A)** Lignin biosynthesis pathway and **(B)** Flavonoid biosynthesis pathway under SAS. Rectangle diagram represents the expression changes of relevant structural genes. The diagram is divided into four equal segments (T1, T2, T3 and T4), each representing the different durations. The vibrant colors of the rectangles symbolize the regulation status of the genes under SAS treatment.

Based on the established phenylpropanoid biosynthesis pathway in model plants, a regulatory network map of rose was constructed. Furthermore, 52 structural genes were identified and their expression profiles based on FPKM values are presented in **Figure 7** (**Supplementary Table S7**). Phenylalanine, serving as a precursor for phenylpropanoid biosynthesis, was produced jointly by the upstream glycolysis pathway and the shikimate pathway, forming a phenylpropane pathway generated in response to early biotic stress under regulation by key rate-limiting enzymes phenylalanine ammonialyase (PAL), cinnamic acid 4-hydroxylase (C4H), and 4-coumarate-CoA ligase (4CL). During the early defense stage, two *PAL*s (Rorug01G0338100 and Rorug03G0164200), one *C4H* (Rorug05G0531000), and one *4CL* (Rorug01G0485400) exhibited significant upregulation at T2.

HCTs are essential for the synthesis of H-, G-, and S-lignin in lignin biosynthesis (**Figure 7A**). Six *HCT*s were identified, with one (Rorug02G0358400) showing significant upregulation after T2. In addition, two key enzymes, cinnamoyl-CoA reductase (CCR) and cinnamyl alcohol dehydrogenase, directly catalyzed the reduction of p-coumaroyl-CoA, generated from the common phenylpropanoid metabolic pathway, to form H-lignin. Eight *CCR*s were identified, among which five *CCR*s (Rorug02G0361500, Rorug07G0275500, Rorug07G0290400, Rorug07G0290700, and Rorug07G0290800) peaked at T2 and one *CCR* (Rorug07G0304000) showed downregulation during SAS. Caffeic acid-O-methyltransferase (COMT) catalyzes the methylation of caffeic acid to synthesize ferulic acid, subsequently promoting sinapic acid accumulation. Two *COMT*s (Rorug01G0477600 and Rorug06G0370500) peaked at T2, while one *COMT* (Rorug07G0083600) demonstrated an upward trend with prolonged SAS duration. Ferulate 5-hydroxylase catalyzes the hydroxylation of intermediates in phenylpropanoid metabolism, such as NADPH^−^ and O^2−^, which reached peak levels at T4.

In the flavonoid biosynthesis pathway (**Figure 7B**), chalcone synthase (CHS) functions as a key enzyme regulating the metabolic direction of flavonoids. Two *CHS*s (Rorug01G0016200 and Rorug01G0016500), one chalcone isomerase (CHI) (Rorug01G0406700), one *F3H* (Rorug02G0125300), and four flavonol synthases (Rorug06G0362900, Rorug06G0363000, Rorug06G0363400, and Rorug06G0363500) demonstrated upregulation, particularly at T2, suggesting that rose exhibits enhanced secondary metabolism of flavonoids in response to SAS. Within the anthocyanin biosynthesis pathway, four dihydroflavonol 4-reductases (DFRs) and one *ANS* were identified, displaying similar expression patterns, with two *DFR*s (Rorug06G0416800 and Rorug06G0416900) and one *ANS* (Rorug07G0180000) showing significant upregulation at T2. The changes in structural gene expression indicate a sophisticated transcriptional regulatory network used by the plant for effective adaptation to SAS.

### 3.7 qRT-PCR validation

**Figure 8:**
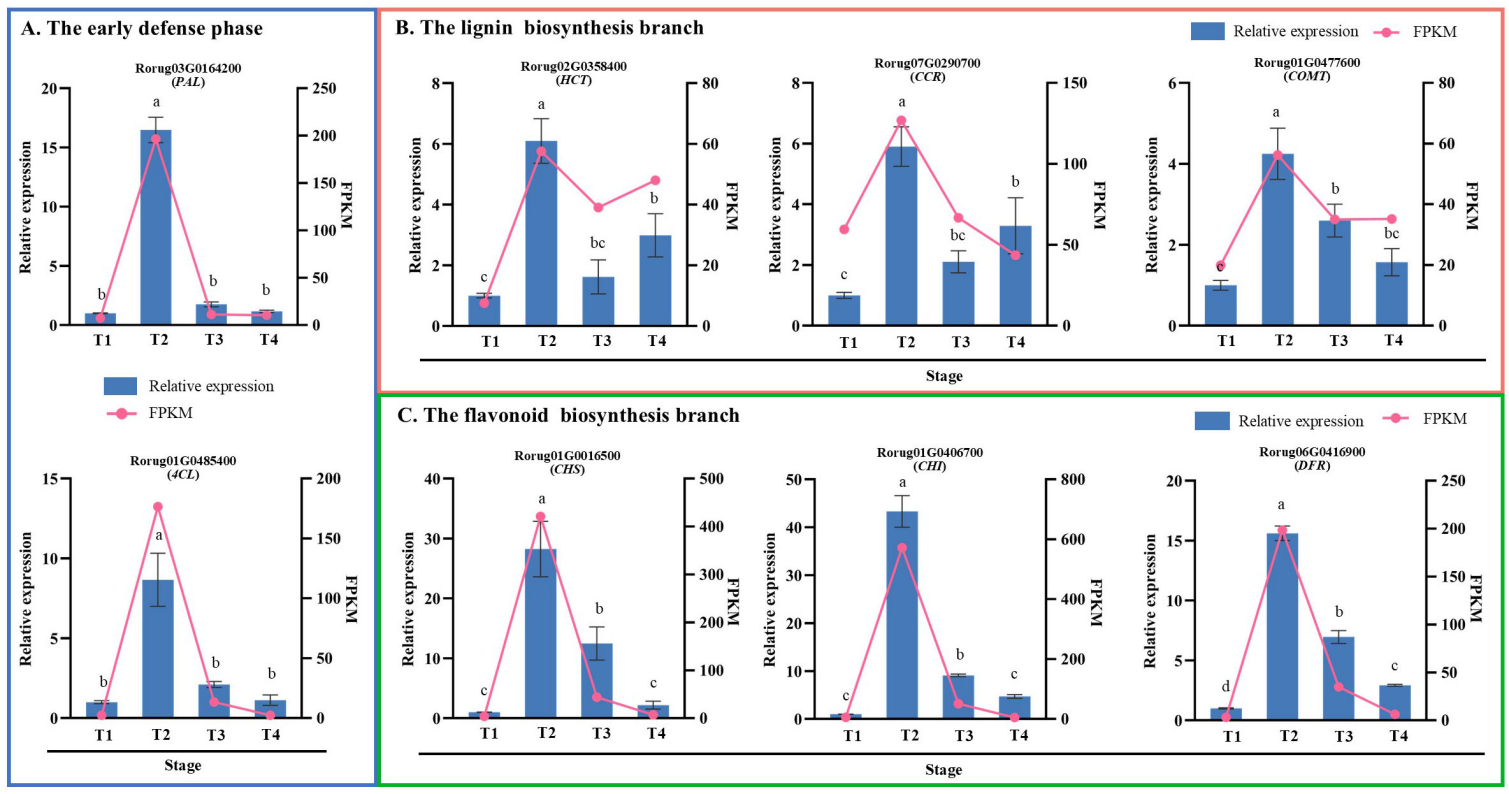
Verification and analysis of eight structure genes expression in phenylpropanoid biosynthesis pathway during the process of SAS by qRT-PCR. Genes were screened from **(A)** the early defense phase, **(B)** lignin biosynthesis and **(C)** flavonoid biosynthesis branch. The left y-axis represents the relative expression levels of genes, normalized using *GAPDH* as the reference gene, while the right y-axis indicates the FPKM values of genes in RNA-seq. For the same parameter, different letters above the bars indicate a significant difference at P<0.05 level.

To verify the reliability of the RNA-seq data, eight structural genes were randomly selected for qRT-PCR analysis, comprising two genes from the early defense stage of the phenylpropanoid biosynthesis pathway and three genes each from the lignin and flavonoid biosynthesis branches. The qRT-PCR detection results demonstrated general consistency with the RNA-seq data trends (**Figure 8**), confirming the accuracy and reliability of the RNA-seq data. Notably, all eight genes exhibited an expression pattern characterized by an initial increase, followed by a decrease, with peak expression occurring at T2 under SAS conditions.

### 3.8 WGCNA analysis

**Figure 9:**
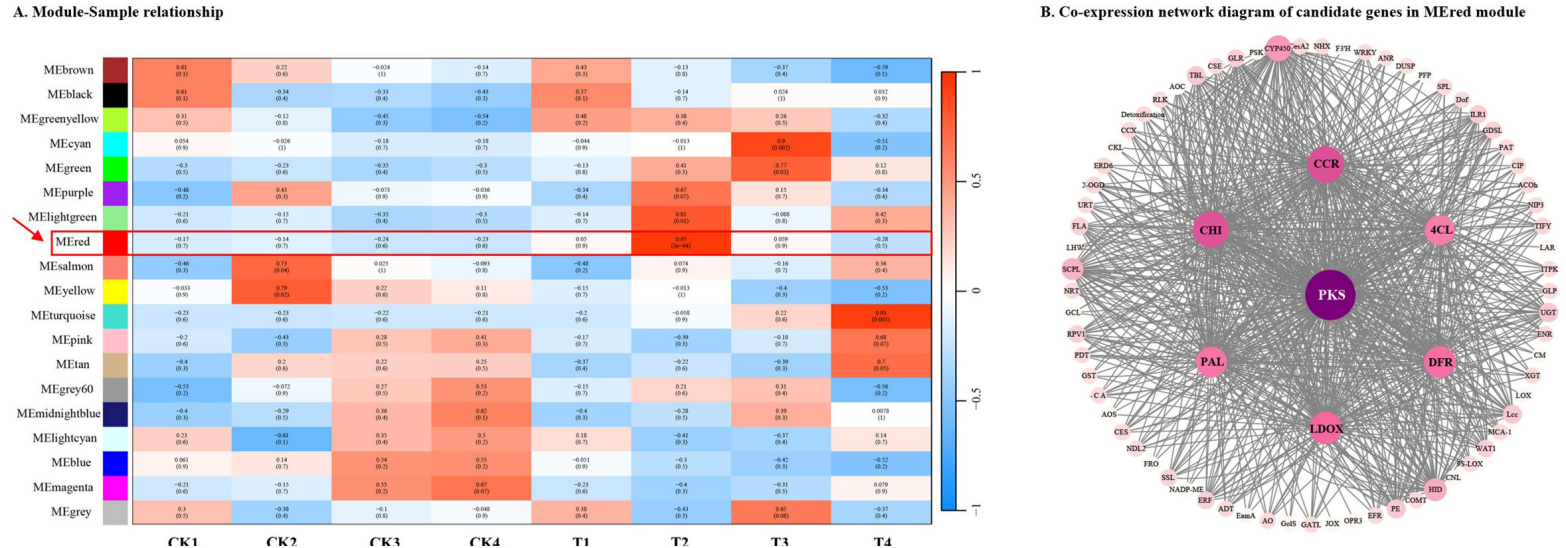
The WGCNA analysis in the process of SAS. **(A)** Heatmap of the correlation between sample and module; **(B)** The co-expressed relationships in the MEred module. The color and area of the circle are determined by the degree value. The higher the degree value, the darker the color and the larger the area of the circle, indicating a stronger co-expression association with other genes.

WGCNA was performed to elucidate the functional genes associated with SAS. The analysis classified all transcripts into 19 modules, with multiple genes in the “phenylpropanoid biosynthesis pathway” clustering in the MEred module (**Figure 9A**). Significantly, *PKS* (polyketide synthase) emerged as a central hub gene, followed by *CHI*, *CCR*, *LDOX* (leucoanthocyanidin dioxygenase), *DFR*, *PAL*, and *4CL*, representing crucial structural genes in the secondary metabolic pathway. The co-expression network incorporated various genes involved in secondary metabolism, including *CYP450* (cytochrome P450), *HID* (2-hydroxyisoflavanone dehydratase), *SCPL* (serine carboxypeptidase-like), and *UGT* (UDP-glycosyltransferase); hormone response-related genes such as *ILR1* (indole acetic acid-amino acid hydrolase), *Acoh* (1-aminocyclopropane-1-carboxylate oxidase homolog), and *CIP* (constitutive photomorphogenic 1-interacting protein); oxidative stress-related genes including *GLR* (glutamate receptor-like), *DUSP* (dual specificity phosphatase), and *GST* (glutathione S-transferase); cell wall development-related genes such as *Lcc* (laccase), *PE* (pectinesterase), *TBL* (trichome birefringence-like), and *FLA* (fasciclin-like arabinogalactan protein); ion transport-related genes including *NHX* (Na^+^/H^+^ exchanger) and *CCR* (cation/calcium exchanger); and several TF genes comprising *ERF*, *WRKY*, *bHLH*, and *TIFY* (**Figure 9B**).

## 4 Discussion

### 4.1 Various physiological indicators actively respond to SAS

Saline–alkali stress, a form of mixed salt stress, disrupts plant cell structure, accelerates ROS production, and leads to tissue damage and metabolic pathway disorders (Vinocur and Altman, 2005; Zhu et al., 2024). In response to accumulated ROS, plants activate antioxidant enzyme systems to reduce cell membrane damage and enhance stress resistance (Gupta and Huang, 2014). In this study, after 12 h of SAS, the accumulated H_2_O_2_ and MDA in rose leaves significantly increased, with DAB and trypan blue staining exhibiting similar patterns (**Figure 1**). These findings indicate that SAS induced ROS production in rose, leading to increased oxidative stress and severe cell membrane damage. In response, the antioxidant enzyme activities, including SOD, POD, and CAT, significantly increased, suggesting ROS damage mitigation through enhanced antioxidant enzyme activities. SOD demonstrated the initial response, indicating its primary role in mediating early ROS responses under mixed SAS, while POD and CAT became more significant in later stages. In plants, antioxidant enzyme activity is precisely regulated by corresponding genes, enhancing ROS scavenging capacity and improving plant tolerance. In *Triticum aestivum*, TaPRX-2A overexpression increases antioxidant enzyme activity, enhances cellular ROS scavenging, and improves salt tolerance in transgenic lines (Su et al., 2020). In *L. perenne*, *CSD* and *FSD* are induced by salt stress, thereby enhancing salinity tolerance (Xu et al., 2022). In *Ipomoea batatas*, *IbCAT2* actively responds to abiotic stresses including drought and salinity (Yong et al., 2017). This study identified two SOD-related genes, eight POD-related genes, and one CAT-related gene, showing high positive correlations with corresponding antioxidant enzyme activities (**Figure 3**). In addition, to address osmotic stress, plants synthesize and accumulate compatible solutes, such as proline, soluble sugars, and soluble proteins, which generate significant osmotic pressure and function as osmolytes during stress (Munns and Tester, 2008; Gong et al., 2020; Munns et al., 2020). Different plants use various substances for osmotic regulation. In *Toona sinensis*, cellular osmotic pressure regulation primarily relies on soluble sugars under salt stress (Liu et al., 2022), while in *A. sativa*, proline and soluble sugars serve as the main osmolytes (Zhu et al., 2020). In this study, the contents of proline, soluble sugars, and soluble proteins showed significant differences under SAS, potentially explaining *R. rugosa*’s high saline–alkali tolerance.

### 4.2 Multiple TFs play crucial roles in responding to SAS

TFs, as primary regulators of stress responses, play a vital role in plant abiotic stress tolerance. Specifically, bHLH, WRKY, APETALA2/ERF, NAC, C2H2, and MYB are involved in responses to multiple abiotic stresses, including salt, drought, heavy metal, and cold (Seo et al., 2015; Ng et al., 2018; Debbarma et al., 2019). In *O. sativa*, salt-responsive ERF1 enhances salt tolerance by amplifying the ROS-activated mitogen-activated protein kinase cascade signal (Schmidt et al., 2013), while OsbZIP18 binds to promoters of key genes in the phenylpropanoid and flavonoid biosynthesis pathways, playing a crucial regulatory role in ultraviolet-B stress response (Liu et al., 2024). WRKY TFs demonstrate significant roles in plant salt stress resistance. Notably, the silencing of *RrWRKY1* in rose increases sensitivity to salt stress, while its overexpression in *Arabidopsis* improves growth indicators under salt stress conditions (Zang et al., 2024). Furthermore, MYB TFs function as both activators and inhibitors of transcription in phenylpropanoid metabolism (Zhou et al., 2009; Bhargava et al., 2010). In *T. aestivum*, *TaMyb1D* acts as a negative regulator of phenylpropanoid metabolism to enhance drought and oxidative stress tolerance (Wei et al., 2017). In *Arabidopsis thaliana*, several MYB TFs, including *MYB28*, *MYB29*, *MYB34*, *MYB51*, and *MYB122*, mediate glucosinolate biosynthesis to enhance stress resistance (Harun et al., 2020). In the present study, statistical analysis of the top 10 TFs revealed that bHLH had the highest proportion in both upregulation and downregulation (**Figure 2**). In *Tritipyrum*, *TtbHLH310* exhibited pronounced sensitivity to salt stress (Li et al., 2025). In *Cucumis sativus*, *CsbHLH041* functions as a key regulator, enhancing salt tolerance in transgenic *Arabidopsis* and cucumber seedlings (Li et al., 2020). Under SAS, numerous bHLHs exercise their transcriptional regulatory functions to enhance rose tolerance, offering new perspectives for investigating molecular mechanisms in SAS response.

### 4.3 Secondary metabolites are accumulated in response to SAS in rose

Plants frequently encounter various biotic and abiotic stresses in natural environments. Throughout evolution, plants have developed mechanisms to integrate diverse environmental signals into their developmental processes, achieving adaptive morphogenesis and precise metabolic pathway regulation. Under stress conditions, while growth is typically inhibited, secondary metabolite production often increases as a protective mechanism. In *Populus alba* × *P. glandulosa*, elevated levels of downstream phenylpropanoid and flavonoid metabolites may be crucial for anthracnose resistance (Xing et al., 2024). In *Paeonia rockii*, glutathione and polyamine pathways play dual roles in enhancing SAS tolerance (Sun et al., 2025). In *Limonium bicolor*, organic soluble compounds and flavonoid accumulation are essential for high salt tolerance (Zhu et al., 2025). In *M. halliana*, photosynthesis levels are maintained during SAS primarily through increased expression of photosynthesis-related proteins (Jia et al., 2019). In the present study, transcriptome data revealed KEGG pathway enrichment in “phenylpropanoid biosynthesis” and “flavonoid biosynthesis” across multiple comparative assessment nodes (**Figure 6**), while GO enrichment of DEGs was associated with “cell wall organization or biogenesis” (**Figure 5**). The phenylpropanoid biosynthesis pathway, a crucial secondary metabolic pathway in plants, regulates plant adaptive growth through metabolites including lignin, flavonoids, and anthocyanins. Lignin, a key product of the phenylpropanoid metabolic pathway, is a major plant cell wall component playing essential roles in cell wall reconstruction and resistance enhancement (Han et al., 2022). Thus, rose resistance to SAS primarily occurs by secondary metabolite accumulation.

### 4.4 Phenylpropanoid biosynthesis pathway responds to SAS actively in rose

The phenylpropanoid biosynthesis pathway is one of the most crucial secondary metabolic pathways in plants. It generates phenolic compounds, such as lignin and flavonoids, through a series of reactions, and these compounds play an essential role in plant stress resistance (Kim et al., 2007; Huang et al., 2009; Zhang et al., 2023). Lignin, a complex phenolic compound, provides structural and defensive barriers for cell walls and is closely associated with plant stress resistance (Liu et al., 2018). Under salt stress, various genes involved in lignin biosynthesis, such as *PAL*, *4CL*, *CCR*, *COMT*, and *HCT*, regulate lignin deposition and secondary cell wall thickening to enhance plant salt tolerance (Hu et al., 2019). Flavonoids, functioning as antioxidants, reduce ROS accumulation caused by stress, thereby mitigating oxidative damage to plants (Jiang et al., 2016). For instance, overexpression of flavonoid biosynthesis genes, such as *CHS*, *F3H*, and *DFR*, enhances the expression of related pathway genes, increases downstream secondary metabolite accumulation, maintains ROS homeostasis, and improves salt resistance during plant development (Kim et al., 2017; Chen et al., 2019; Zhang et al., 2024). Type III PKSs are widely distributed in plants and have been identified in various angiosperms (Kumar and Ellis, 2003; Lussier et al., 2012; Pothiraj et al., 2021). Research has primarily focused on two major branches of phenylpropanoid metabolism and biosynthesis: flavonoids and lignin. In pear, *PbPKS* determines fruit quality by participating in stone cell and lignin synthesis (Li et al., 2018). In this study, during SAS exposure at 150 mmol·L^−1^, multiple genes in the phenylpropanoid biosynthesis pathway were significantly activated (**Figure 7**). The *PAL*, *4CL*, *HCT*, *CCR*, *COMT*, *CHS*, *CHI*, and *DFR* genes were screened and validated by qRT-PCR, revealing that these genes exhibited an initial increase, followed by a decrease, peaking at T2 (**Figure 8**). *PKS*, functioning as a hub gene, occupied a central position in WGCNA (**Figure 9**). Thus, in the early stages of SAS, rose primarily responds to stress through dynamic gene transcription, subsequently regulating secondary metabolite accumulation to cope with SAS.

## 5 Conclusions

**Figure 10:**
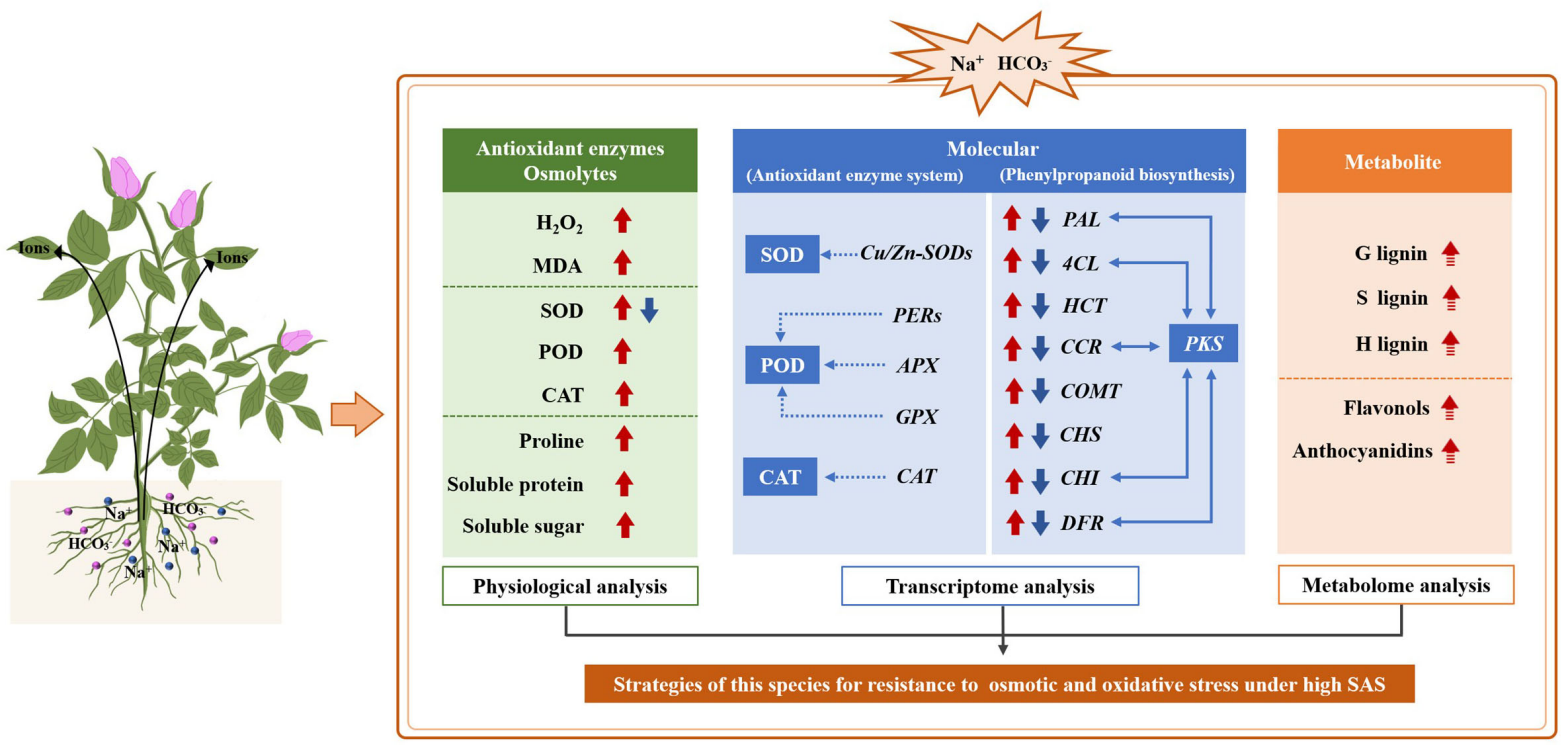
Model of the regulatory mechanisms of *Rosa rugosa* in response to salt-alkali treatments. The one-way arrows (short and thick) represent expression patterns, where red and blue arrows denote upregulation and downregulation, respectively. The simultaneous presence of red and blue arrows indicates a trend of first rising and then falling. The one-way arrows (long and thin) represent the regulatory relationship between genes and enzymes. The two-way arrows represent a strong co-expression relationship between genes. The solid arrows indicate verified results, while dashed arrows denote speculated results.

SAS altered the physiological, biochemical and molecular processes, thereby affecting the growth of in *R. rugosa*. A conceptual model was proposed to clarified the regulatory network of salt-alkali tolerance (**Figure 10**). In this model, SAS exacerbated oxidative damage to its cell membranes, which counteracted the stress via continuously accumulating proline, soluble protein and soluble sugar and persistently enhancing SOD, POD and CAT activity. Correspondingly, six *PERs*, one *APX* and one *GPX* were screened, which potentially associated with POD activity; one *CAT* was identified that may correlate with CAT activity, and one *CSD* was found that could be related to SOD activity. In addition, the “Phenylpropanoid biosynthesis” pathway a significant role in response to the oxidative and osmotic stress induced by SAS. Multiple key structural genes in the pathway, such as *PAL*, *4CL*, *HCT*, *CCR*, *COMT*, *CHS*, *CHI* and *DFR*, exhibited an expression trend of first rising and then declining during SAS, and *PKS* is highly likely to be the hub gene in the pathway. Ultimately, we speculate that rose accumulate the end products of the “phenylpropanoid biosynthesis” pathway, such as lignins, flavonols and anthocyanidins, serves as a defense mechanism against SAS. This model illustrates the species’ strategies for resisting osmotic and oxidative stress and regulating secondary metabolism under high SAS from multiple perspectives. Furthermore, the results provide a theoretical basis for understanding SAS response mechanisms in rose and offer new directions for breeding rose varieties with enhanced saline–alkali tolerance.

## Data Availability

The data presented in the study are deposited in the NCBI repository, accession number PRJNA1300623.
