# Supplementary figures and images for: Integrated physiological and transcriptomic analysis reveals the key pathways of Rosa rugosa in response to salt-alkali stress
Source: Front Plant Sci. 2025 Dec 1;16:1679259. doi: 10.3389/fpls.2025.1679259 (PMC12702943; doi:10.3389/fpls.2025.1679259)

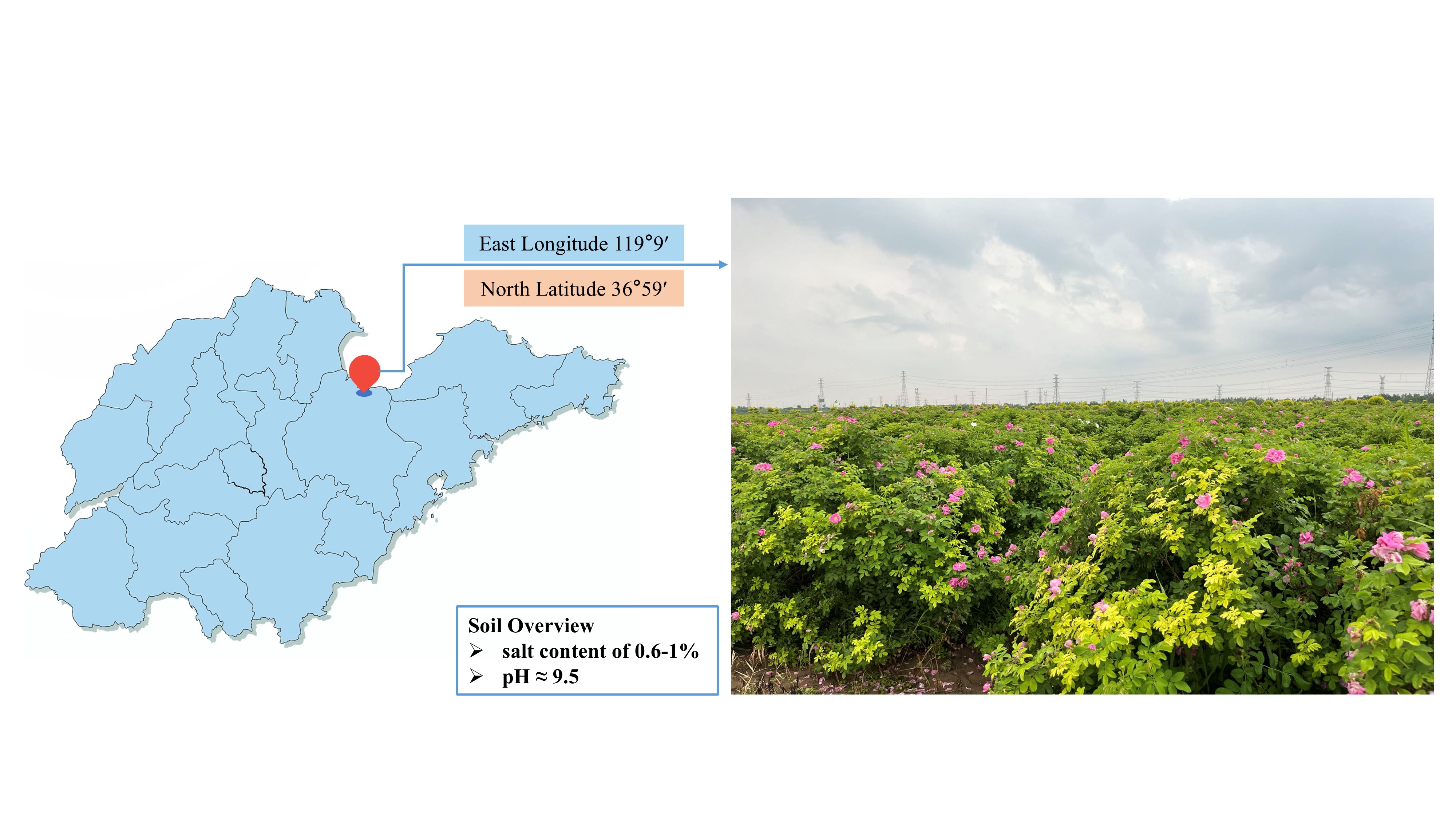

Supplement: Supplementary file 1 [file Image1.jpeg]

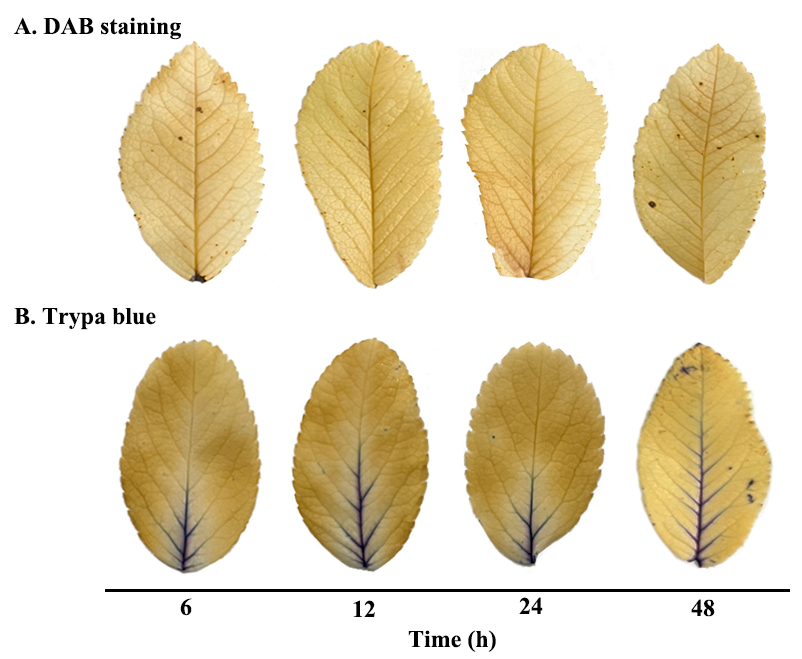

Supplement: Supplementary file 2 [file Image2.jpeg]
